# Model of Host-Pathogen Interaction Dynamics Links *In Vivo* Optical Imaging and Immune Responses

**DOI:** 10.1128/IAI.00606-16

**Published:** 2016-12-29

**Authors:** Angelique Ale, Valerie F. Crepin, James W. Collins, Nicholas Constantinou, Maryam Habibzay, Ann C. Babtie, Gad Frankel, Michael P. H. Stumpf

**Affiliations:** a Department of Life Sciences, Centre for Integrative Systems Biology, Imperial College London, London, United Kingdom; b Department of Life Sciences, Medical Research Council (MRC) Centre for Molecular Bacteriology and Infection, Imperial College London, London, United Kingdom; University of California, Davis

**Keywords:** antibiotic therapy, bioluminescent bacteria, Citrobacter rodentium, dynamic modeling, *espO* mutant, *in vivo* imaging

## Abstract

Tracking disease progression *in vivo* is essential for the development of treatments against bacterial infection. Optical imaging has become a central tool for *in vivo* tracking of bacterial population development and therapeutic response. For a precise understanding of *in vivo* imaging results in terms of disease mechanisms derived from detailed postmortem observations, however, a link between the two is needed. Here, we develop a model that provides that link for the investigation of Citrobacter rodentium infection, a mouse model for enteropathogenic Escherichia coli (EPEC). We connect *in vivo* disease progression of C57BL/6 mice infected with bioluminescent bacteria, imaged using optical tomography and X-ray computed tomography, to postmortem measurements of colonic immune cell infiltration. We use the model to explore changes to both the host immune response and the bacteria and to evaluate the response to antibiotic treatment. The developed model serves as a novel tool for the identification and development of new therapeutic interventions.

## INTRODUCTION

Enteropathogenic Escherichia coli (EPEC) is a major cause of infantile diarrhea and mortality in low-income countries (1). The disease progress of EPEC can be studied in mice using the mouse-specific pathogen Citrobacter rodentium, which mimics the human course of infection. Following oral inoculation, C. rodentium first colonizes the cecum before the pathogen disseminates to the distal colon. The pathogen uses type 3 secretion system (T3SS) effectors for colonization, evasion of host immune responses, including inhibition of NF-κB signaling and inflammatory caspases (2), and inhibition of intrinsic and extrinsic apoptosis (3–5). The infection peaks between 7 and 9 days postinfection (p.i.) and plateaus for a few days before being cleared by 18 to 21 days postinfection. Clearance of C. rodentium is mediated by robust inflammation, which includes recruitment of immune cells (including neutrophils as well as Th-22 and Th-17 CD4^+^ T and B cells) (6), production of antimicrobial peptides (7, 8), and competition from the microbiota (9–12).

Infection with C. rodentium has been studied extensively using postmortem analyses (6). The recent development of bioluminescent bacteria that emit visible light (13–15) has enabled studying disease progression also *in vivo* using optical imaging. Optical imaging has been emerging as a versatile tool to study disease progression in small animals *in vivo* (16). Using bioluminescent or fluorescent markers, cells and substances can be tracked across the whole body (17). More and more research groups are acquiring this state-of-the-art technique in-house to complement the traditional postmortem techniques that provide information on disease parameters on a more detailed level but only at distinct time points and locations. Using a system for combined bioluminescence and X-ray computed tomography (CT) imaging, bacterial burden can be quantified and localized with precision.

However, in order to enable the interpretation of *in vivo* images in terms of postmortem-derived disease parameters, a model that links whole-body *in vivo* imaging results to cellular and molecular data obtained postmortem, not all of which can be visualized, is crucial.

A model of the underlying biological processes that give rise to the signals that are measured with whole-body imaging can place processes occurring at the cellular or molecular scale in the context of processes taking place over a much larger scale, in different parts of the animal, or at different time points during the course of disease. In other contexts, such as in the investigation of inflammatory bowel disease, detailed models of host-pathogen interaction in the gut have been described (18, 19). In this study, we develop a model of C. rodentium infection to create a direct link between state-of-the-art *in vivo* whole-body imaging results and detailed biological knowledge at the cellular scale. We show how the model can be used to analyze changes in host immune response, to study mutations in the pathogen, and to analyze and simulate the response to antibiotic treatment.

## RESULTS

### Dynamics of C. rodentium colonization, clearance, and recruitment of immune cells.

We first establish the general dynamics of host-pathogen interaction during C. rodentium infection. These results are used to determine the model, to link experimental *postmortem* data to results from *in vivo* imaging, and to simulate the contribution of host immunity to bacterial clearance.

**FIG 1:**
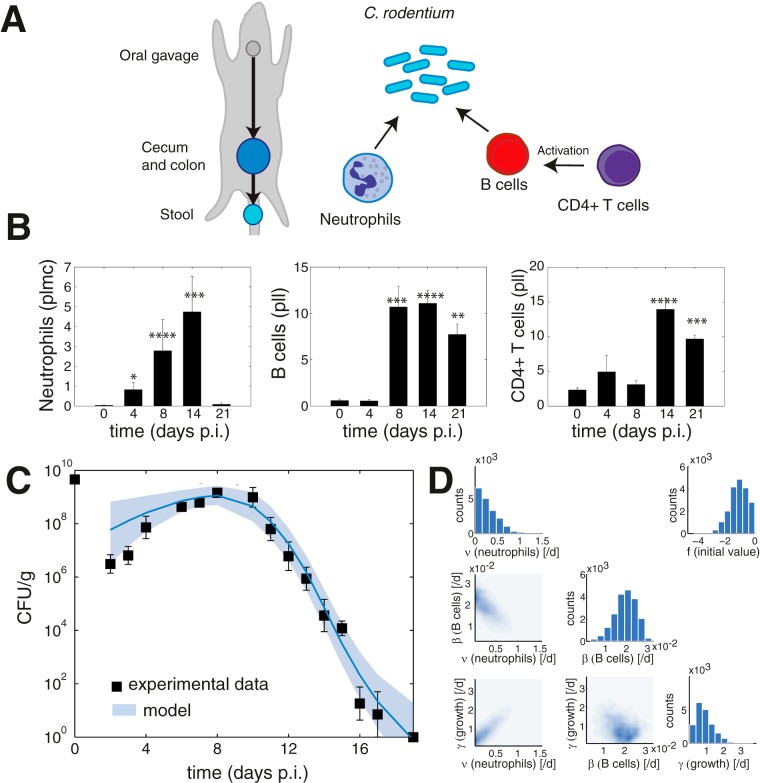
Dynamics of wild-type C. rodentium infection. (A) Model schematic. After oral gavage, C. rodentium colonizes the cecum and colonic mucosa and is shed in the stool. C. rodentium infection is cleared mainly by neutrophils and B cells activated by CD4^+^ T cells. (B) Immune cell data from colon samples. Neutrophils are shown as a percentage of live myeloid cells (plmc), and B cells and CD4^+^ T cells are depicted as percentages of live lymphocytes (pll). (C) Fit of the model to the wild-type data. Experimental data (squares), mean model estimate (line), and 90% confidence interval were calculated with the model (shaded area). (D) Model parameter estimates. The histograms show which values were accepted for each of the parameters (marginal posterior parameter density). Higher counts indicate we found more good fits with parameter combinations that include those parameter values. The scatter plots show the correlation between two of the estimated parameters (pairwise posterior parameter density). Dark blue areas indicate regions with a large number of accepted parameter combinations. The scatter plot of neutrophil parameters against B cell parameters shows that combinations of small values for the neutrophil elimination rate and large values for the B cell elimination rate and vice versa resulted in good fits of the model to the data (negative correlation). The scatter plot of neutrophil parameters against growth parameters shows that combinations of small values for the neutrophil elimination rate and small values of the growth rate, as well as larger values for the neutrophil elimination rate and larger values of the growth rate, resulted in good fits of the model to the data (positive correlation).

Following oral administration, C. rodentium colonizes the cecum and colon of mice (Fig. 1A). Bacteria are continually shed into the feces, and the level of bacteria in the stool correlates with the burden of attached bacteria in the colon (6). We measured the bacterial levels in the stool for wild-type C57BL/6 mice over the course of infection (Fig. 1C); the peak of infection is at day 8 postinoculation, and the infection is cleared at around day 21. Clearance of C. rodentium is achieved through a combination of innate and adaptive defense mechanisms and competition by the microbiota. Neutrophils, B cells, and T cells play a major role in the elimination of the pathogen (6). We measured recruitment of immune cells to colonic samples obtained postmortem (Fig. 1B). Neutrophils start accumulating in the colon at the early stage infection, followed by B cells at a later stage; CD4^+^ T cells are detected from day 14 postinfection.

We modeled how a change in bacterial shedding depends on the host immune response. We included neutrophils and antibody-producing B cells as elimination factors, and we accounted for their dependence on the presence of CD4^+^ T cells. We relied on the following assumptions: elimination of bacteria by neutrophils is proportional to the measured levels of neutrophils, and B cells first need to be activated before they are effective; B cells are activated by CD4^+^ T cells; and elimination through the antibody response mediated by B cells is proportional to the levels of active B cells. We further assume that the T cell population only effectively activates B cells after day 8.

Under these assumptions, we model the change in bacterial levels, *C*, in the colon over time (*dC*/*dt*) by the simple equation *dC*/*dt* = γ · *C* − ν · *N* · *C* − β · *B* · CD4^+^ T · *C* − θ · *C*. The positive growth term (γ · *C*) results in an increase in the bacterial load over time, and the negative elimination terms (neutrophils [ν · *N* · *C*] and B cells [β · *B* · CD4^+^ T · *C*]) and the negative treatment term (θ · *C*) result in decreases of the bacterial load over time. The growth parameter, γ, represents the bacterial replication rate per day, and the parameters ν and β represent bacterial elimination rates per day per active immune cell population. *N* is the concentration of neutrophils, *B* is the concentration of B cells, and CD4^+^ T is the concentration of CD4^+^ T cells. The last term in the model corresponds to elimination as a result of treatment. Initially we set the treatment parameter, θ, to zero, but we will use this term later. We modeled the initial value of the level of bacteria in the stool by *C*_0_ *= fC*_O_, where *C*_O_ is the concentration of bacteria during oral gavage and the parameter *f* represents the fraction that is present in the colon at day 2 of the infection.

The *in vivo* dynamics of this system have not previously been modeled, and therefore we lack reliable estimates of model parameters (such as rates of killing of bacteria due to different parts of the immune system). We therefore used the data generated here to obtain estimates of the parameters. We used a method that results in not just one point estimate of the parameters, which would only correspond to one good fit of the model to the data, but instead generates multiple probable parameter estimates that all result in good model fits. This provides an estimate of the uncertainty in the parameters, which may be due to biological variability or noise in the data (i.e., we infer parameters in a Bayesian setting) (20). We obtain estimates for the parameters by fitting the model to measurements of the bacterial load in the stool, obtained from wild-type C57/BL6 mice infected with wild-type C. rodentium, assuming a direct correlation between the levels of attached bacteria in the colon and bacteria in the stool (Fig. 1C and D). We used stool data to calibrate the model instead of imaging data, as stool data are more abundantly available. In the next section we will link the model to *in vivo* imaging data of the bacterial load in the abdomen.

We used the measured immune cell infiltration in the model as the input (N, B, and CD4^+^ T cells). We estimated the immune cell infiltration for time points between measured time points using linear interpolation. To estimate parameters, we used an algorithm (the Bayesian inference algorithm Metropolis-Hastings) which allowed us to obtain multiple probable estimates for parameter values (we generated samples from the posterior parameter distribution). This is a sequential method that starts with some initial parameter estimates (sampled from prior parameter distributions that are defined according to our current knowledge about the parameter values) and iteratively updates these estimates based on how well model simulations using the sampled parameter values fit the data (21, 22). Whether the estimate of the bacterial load is close to the measured CFU per gram was determined by comparing it to the measured variance in the bacterial levels at each time point (i.e., a Gaussian likelihood). We used this set of parameter estimates to simulate trajectories of bacterial concentrations. Figure 1C shows the mean and the interval that includes 90% of the modeled bacterial loads (i.e., the 90% confidence interval) calculated from these simulated trajectories, which closely matched the measured trajectory of bacterial load.

The model allows us to measure the relative impact of the immune cell populations. In particular, the negative correlation between ν and β shows that a decrease in the rate of elimination by neutrophils is compensated for by an increase in the rate of elimination by B cells. Furthermore, we find that the rate of elimination by neutrophils increases when the bacterial growth rate is higher (positive correlation between γ and ν) (Fig. 1D).

### *In vivo* bioluminescence imaging data.

**FIG 2:**
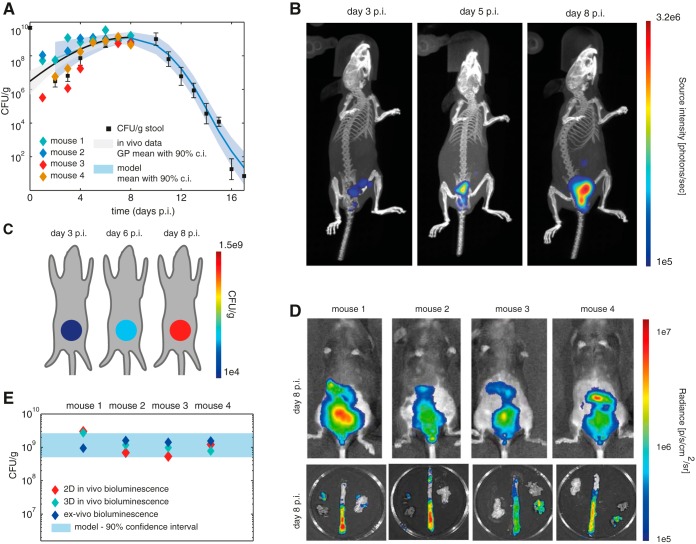
*In vivo* bioluminescence imaging data. (A) Quantitative comparison of *in vivo* whole-body bioluminescence with CFU/g stool for wild-type C57/BL6 mice. (Stool data from Fig. 1C are displayed here for comparison.) (B) 3D *in vivo* imaging results for a representative mouse. (C) Modeled bacterial load corresponding to the *in vivo* experiment. (D) 2D bioluminescence imaging of 4 mice and corresponding bioluminescence imaging results of mouse intestines *ex vivo*. (E) Comparison of quantification results for 2D, 3D, and *ex vivo* bioluminescence measurements and the 90% confidence interval (c.i.) estimated with the model.

We linked the model results to longitudinal whole-body C. rodentium imaging *in vivo*. We measured disease progression in four mice infected with bioluminescent C. rodentium using the IVIS system for optical tomography and X-ray CT from day 1 to the peak of infection at day 8. Reconstruction of the three-dimensional (3D) bioluminescence distribution for a representative mouse clearly shows a local increase in bioluminescence signal in the abdomen (Fig. 2B). We quantify the bacterial load by summing the total bioluminescence signal in the abdomen for each mouse at each time point (Fig. 2A). For the purpose of comparison with model results, we normalized these signals to the one obtained at day 8. We determined the trend in the means and standard deviations of the quantified imaging data using a method that assumes a certain smoothness over time and for which no additional parameters are necessary (we use Gaussian process [GP] regression, a nonparametric Bayesian method for nonlinear regression [23, 24]). The trends apparent in the quantified *in vivo* imaging data correspond nicely to the confidence interval calculated with the dynamic model (Fig. 2A).

Using the model, we predicted the outcome of imaging experiments. In Fig. 2C we show the estimated mean bacterial loads on three different days. In this way, the model can serve as a tool for experimental design (25, 26) and to monitor imaging outcomes against predictions during the course of an ongoing experiment.

For the same four mice, we also acquired the 2D bioluminescence distribution at day 8 and the *ex vivo* 2D bioluminescence distribution of the mouse intestines (Fig. 2D). We quantified the signal in these images in the same way as that described above and compared quantification across the three acquisition methods (Fig. 2D and E). The quantified signals correspond to the predicted confidence interval.

### Perturbations in the host immune response.

**FIG 3:**
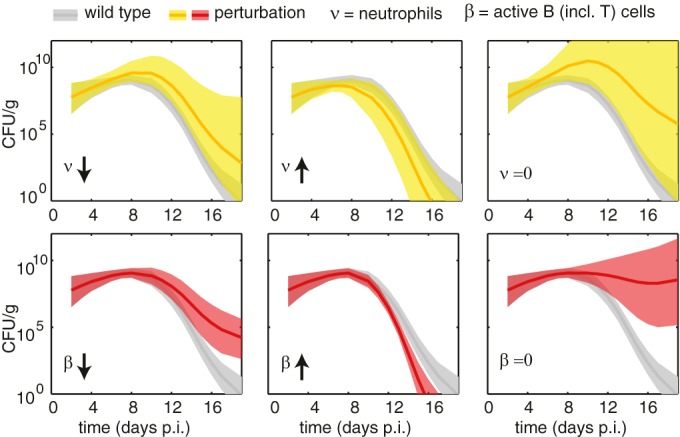
Perturbations to the host immune response. Shown are the simulated bacterial loads in response to variations of the model parameters to mimic changes in the immune response. We decreased the parameter estimates by 50% (arrow down), increased the estimates by 50% (arrow up), or set them to zero (=0). This simulates an increase or decrease in the elimination rate of bacteria by the immune cell population or a knockout of the immune cell population by setting estimates to zero. (Top) Variation of estimated parameter distribution corresponding to the neutrophil concentration (ν). (Bottom) Variation of the parameter distribution estimated for the active B cell concentration (β). Gray-shaded areas indicate the 90% confidence interval estimated for wild-type mice. Yellow-shaded areas indicate 90% confidence intervals for the simulated trajectories in response to perturbations to the neutrophil parameters. Red-shaded areas indicate 90% confidence intervals for simulations corresponding to variations in B cell parameter distributions. Boldface lines show the mean simulated trajectories.

Changes in the host immune cell response, as observed, for example, when using knockout mice, can result in a change in bacterial death rate due to altered immune system activity. We investigated the effects different cells of the immune system have by the model parameters corresponding to the activity of neutrophils (ν) and B cells (β), dissecting the contributions of these different immune cell populations (Fig. 3). We either increased parameters by 50%, decreased them by 50%, or set them to zero, and we compared the resulting model simulations.

A change in the neutrophil term (Fig. 3, top row) has an impact on the height of the peak of infection: even a small decrease in neutrophil effectiveness may lead to inability to clear the infection. A change in the B cell term (Fig. 3, bottom row), being active during the later stage of disease progression, has a less pronounced effect. Setting activity of either cell type to zero would lead to inability to clear the infection.

The perturbations considered here can be related to knockout mice described in the literature. A decrease in the accumulation of neutrophils and macrophages to the site of infection has been observed in MyD88^−/−^ mutant mice. These mice lack MyD88, which is part of the signaling pathway of Toll-like receptors that activates inflammatory cytokine production, and as a result the recruitment of neutrophils and macrophages to the site of infection is impaired (27). Bacterial levels quickly expanded followed by death from day 7 when the level of colonizing bacteria reached high levels. A similar quick expansion toward high levels of bacterial burden is also observed in the corresponding simulation (Fig. 3, top right image).

Setting both B cells and the T cell population to zero (Fig. 3, bottom right image) is related to *rag1*-deficient mutant mice. Infection in this mouse model experimentally becomes a chronic infection (28). The corresponding simulation shows a similar pattern of chronic infection, i.e., the immune response does not clear the infection over the time scales considered here (or in the experiments).

### Immune response to infection with C. rodentium *espO* deletion mutant.

**FIG 4:**
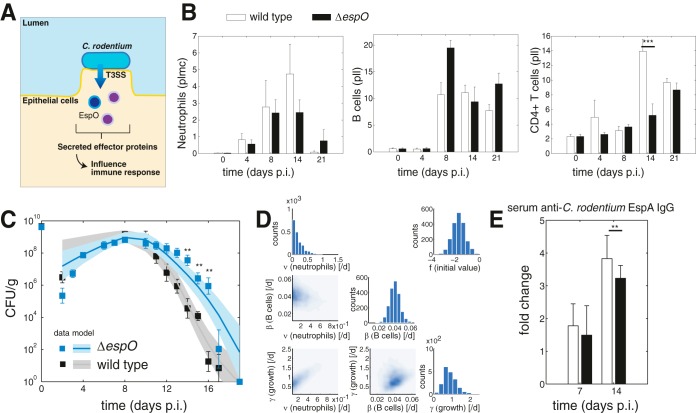
Analysis of infection by C. rodentium Δ*espO* mutant. (A) Schematic. EspO is a T3SS effector injected by C. rodentium into host colonic epithelial cells and influencing immune responses. (B) Immune cell data from colon samples. Neutrophils are shown as a percentage of live myeloid cells (pllm), and B cells and CD4^+^ T cells are depicted as percentages of live lymphocytes (pll). (C) Model fit to experimental data of bacterial load in the stool. (Wild-type stool data from Fig. 1C are displayed here for comparison.) (D) Model parameter estimates. The histograms show which values were accepted for each of the parameters (marginal posterior parameter density), with higher counts indicating we found more good fits with parameter combinations that include those parameter values. The scatter plots show the correlation between two of the estimated parameters (pairwise posterior parameter density). Dark blue areas indicate regions with a large number of accepted parameter combinations. (E) Experimental ELISA measurements of serum anti-C. rodentium EspA IgG.

We next used the model to investigate changes in the immune response when wild-type C57BL/6 mice are infected with C. rodentium carrying a deletion of the T3SS effector EspO, a homologue of OspE from Shigella, shown to stabilize focal adhesion and block cell detachment (29, 30) (Fig. 4A).

We measured the levels of neutrophils, B cells, and CD4^+^ T cells in colonic tissue samples at four time points, as well as the level of bacterial shedding in the stool at 14 time points, spanning the course of infection. For the *espO* mutant, we observed a significantly higher bacterial load at days 14, 15, and 16 postinfection compared to infection with wild-type C. rodentium. The recruitment of immune cells to the colon following infection with the mutant was generally similar to what we observed during wild-type infection (Fig. 4B), with the main difference being that T cell infiltration was reduced on day 14.

We then further analyzed the dynamics by fitting our model against C. rodentium Δ*espO* stool measurements to characterize the response dynamics in this mutant (estimating parameter values in the same way as was described above), using the immune cell populations measured for the mutant as the starting point for the analysis (Fig. 4B). We found that the estimated parameter correlations are similar to the parameters we obtained when fitting the wild-type infection dynamics, and that the order of magnitude of the estimated parameters was the same (Fig. 4C and D).

Elimination due to the B cell term in the model depends on B cell abundance, their activation by CD4^+^ T cells, and the estimated rate parameter. At day 14, the total elimination rate corresponding to the active B cell term in the model was smaller during infection with the Δ*espO* mutant than with the wild type, mainly due to the difference in T cell abundance. This provides a possible explanation for the prolonged high bacterial load during infection with the Δ*espO* mutant.

In order to validate this finding, we proceeded to measure the level of antibodies against EspA experimentally following wild-type and Δ*espO* mutant infections (Fig. 4E), and we found a significantly lower level of C. rodentium-specific antibodies at day 14 during infection with the Δ*espO* mutant, confirming the model prediction.

### Therapeutic response.

**FIG 5:**
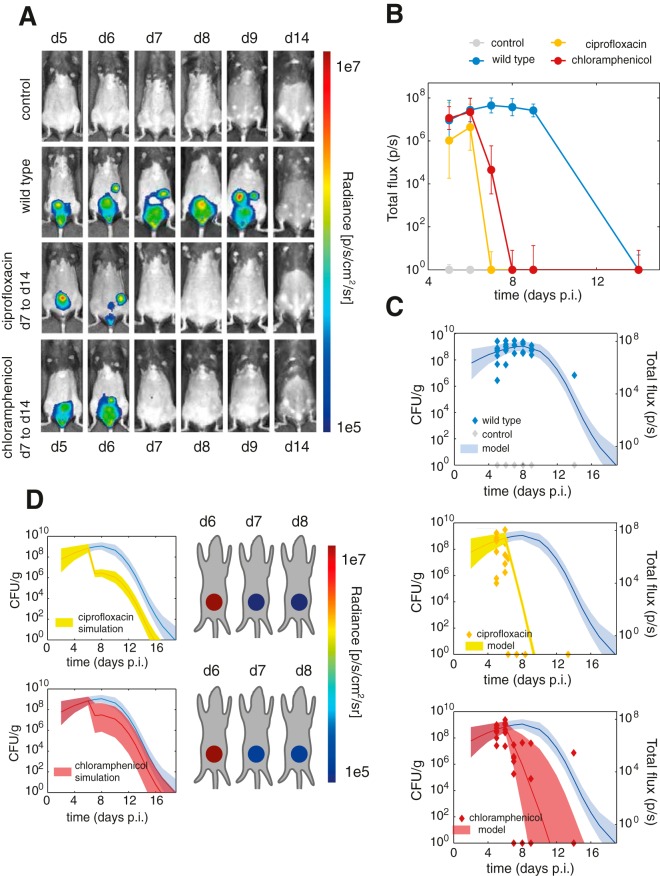
Evaluation of antibiotic treatment. (A) 2D imaging results of uninfected mice, infected mice, infected mice that received ciprofloxacin treatment started after the day 6 (d6) measurement to day 14, and infected mice that received chloramphenicol treatment during the same period. (B) Quantified 2D imaging results. (C) Model results compared to quantified imaging results. (D) Simulated response to treatment with ciprofloxacin or chloramphenicol for 1 day started after the day 6 measurement of bacterial load.

Finally, probing a pivotal aspect of bacterial infection biology, we used the calibrated model to investigate the therapeutic response to two antibiotic treatments. We evaluated antibiotic treatment of C. rodentium infection with the bactericidal antibiotic ciprofloxacin and with bacteriostatic chloramphenicol. We measured the C. rodentium bacterial burden *in vivo* during antibiotic chemotherapy using 2D bioluminescence imaging between days 6 and 14 postinfection (Fig. 5A). Treatment was started on day 6 postinfection and continued until the end of the experiment. We quantified the imaging data by summing the signal (total flux) from the abdomen for each mouse (Fig. 5B), corrected for a constant background signal level of 1e5 photons/s, determined by quantifying the uninfected mouse images. The measured bacterial loads showed a sharp decrease when treatment with either antibiotic was started, while the standard deviation was larger during treatment with chloramphenicol, suggesting more variable therapeutic effectiveness. The quantified 2D imaging data of wild-type infected mice show excellent correspondence to the calibrated model (Fig. 5C, first image).

We simulated treatment with ciprofloxacin by including the treatment term in the model (θ), resulting in a steep decrease of the bacterial load, with a narrow confidence interval (Fig. 5C, second image). We modeled treatment with chloramphenicol by changing the growth term in the model (γ). The bacterial growth rate is a net rate of bacterial multiplication and death, and stopping multiplication would make this term negative. This results in a decrease in the bacterial load, but the progression is now more variable than it would be if the treatment resulted in the direct killing of bacteria (Fig. 5C, third image).

Finally, we can use the model for experimental design (25, 26) to determine the effect of treatment during a specific time window. We can evaluate any combination of starting and stopping times for the treatment by simulating with the model in order to identify which would be the most relevant experiments to carry out. We simulated treatment with a single dose of chloramphenicol and ciprofloxacin (at day 6) and found that compared to treatment with a single dose of chloramphenicol, treatment with a single dose of ciprofloxacin would have a larger beneficial effect (Fig. 5D).

## DISCUSSION

In this study, we developed a mechanistic modeling approach that links bacterial colonization levels measured *in vivo* to the immune response, including possible treatments. Whole-body bacterial colonization was measured in the stool and observed *in vivo*, and immune cell populations (neutrophils, CD4^+^ T cells, and B cells) in the gastrointestinal tract were obtained postmortem. The modeled colonization levels correspond very well to the colonization levels observed with both 2D- and 3D-quantified *in vivo* imaging data. We used the mechanistic model to unravel the effect of each cell type, as well as different antibiotic treatments, on the course of disease.

By simulating the outcomes corresponding to observed changes in host immune response due to mutations described in the literature using our dynamic model, we analyzed a range of separate literature studies within a single modeling framework. The model allows us to draw conclusions regarding the role of each of the aspects of the immune response in infection clearance in wild-type (C57BL/6) mice. Moreover, the results obtained from wild-type mice suggest that the sometimes disparate behavior found in different experimental studies can be combined into a clear picture of infection dynamics with the help of the general model presented.

Second, we have analyzed the immune response to the C. rodentium Δ*espO* mutant and found prolonged colonization compared to the wild type. Using the model, we then predicted that a lower total elimination rate of B cells at day 14 could explain the prolonged infection, which we validated experimentally by measurement of IgG antibodies against EspA. This shows how to use the model to identify gaps in our knowledge that require further investigation. Such successful predictions also serve to validate the model. The success of the current study opens the prospect of using a similar approach to analyze the influence of other effectors on the immune response, such as Tir, which can affect the recruitment of neutrophils (31).

Finally, we have used the dynamic model to evaluate the response to antibiotics, showing that simulations with the model accurately describe two different effects: the effect of antibiotics that directly kill bacteria and the effect of antibiotics that stop growth. Comparison with experiments shows that the model captures the variation of colonization levels in time both qualitatively and quantitatively. Therefore, these types of simulations can be used as a tool for experimental design and to compare imaging results with expectations during imaging experiments. The simulations also allow feasibility studies prior to experiments to indicate whether the experiments would result in a detectable change in the bacterial load *in vivo*, for example, in the context of probiotic experiments (32), and to explore different treatment regimens in terms of timing and duration.

What is already becoming clear from the current study is that the use of bacteriostatic antibiotics risks that there remains for a considerable time, probably longer than a typical or practical therapeutic time course, a pool of bacteria that are ready to lead up to recurrent disease, persistence, and the evolution of antibiotic resistance. Here, modeling of the type developed in the current study offers a rational way to prioritize therapeutic approaches.

The modeling approach that we provide in this study can be applied to a wide range of experimental studies. In this work we have used quantified bioluminescence data, but the same type of model can be used in combination with other state-of-the-art optical imaging modalities, such as fluorescence optical tomography and X-ray CT (33). The emerging range of markers that is being developed for visualizing many different disease parameters *in vivo* strongly increases the number of links that can be made between *in vivo* and postmortem observations. Of particular interest are markers that visualize the species included in the model *in vivo*, such as markers for neutrophils and T cells.

Previous studies have also combined *in vivo* imaging and postmortem experiments to analyze C. rodentium infection. For example, Hall et al. (15) have studied the role of natural killer cells during C. rodentium infection using *in vivo* optical imaging, and Cronin et al. (34) have studied the use of C. rodentium as a vehicle for cancer treatment. The modeling approach that we describe here is ideally suitable to relate the reported *in vivo* colonization levels to the postmortem-derived parameters in these studies to provide a direct connection. Compared to available simulation platforms (19), the limited number of host-pathogen interactions included in our model makes it specific to the experimental setup, enabling us to directly validate theoretical results against experiments. More detailed spatial information can be included using a model with multiple compartments, and different scale levels can be included by combining this with agent-based modeling, as has been done for investigating tuberculosis (35). However, the spatial distribution of bacteria would be required, for example, by visualizing this *in vivo* using X-ray CT contrast agents.

The relatively simple modeling approach that we employ, using an ordinary differential equation (ODE) model in combination with Bayesian parameter inference, facilitates the interaction between theory and experiment and enables combined research toward the discovery of major biological breakthroughs. In this way, the developed modeling framework serves as an interface between theory and experiment and can play an important role in the future for specifically identifying gaps in our current knowledge, to define key points that require further investigation, both in terms of modeling and experiment, as well as for the development of suitable treatments.

## MATERIALS AND METHODS

### Bacterial strains and growth conditions.

The bacterial strains and primers used in this study are listed in Table 1. Bacteria were grown in Luria-Bertani (LB) medium supplemented with kanamycin (50 mg ml^−1^) and nalidixic acid (50 mg ml^−1^) as required.

**TABLE 1:** Strains and primers used in this study

Strain, plasmid, or primer	Description/nucleotide sequence (5′ to 3′)	Reference or source
Strains		
ICC169	Wild-type C. rodentium O152 serotype	36
ICC180	Bioluminescent C. rodentium O152 serotype	36
ICC1333	C. rodentium espO deletion mutant, KanR	This study
Plasmids		
pKD46	Coding for the lambda Red recombinase	37
pKD4	Coding for the kanamycin resistance cassette	37
pICC929	pET28a expressing C. rodentium EspA with a C-terminal 6-histidine tag	This study
Primers		
EspO(W77A)-Fw	GCCTCAGAAGCTCAGGCGATGTGCAAAATTATAG	
EspO(W77A)-Rv	CTATAATTTTGCACATCGCCTGAGCTTCTGAGGC	
k1-EspO-del-Fw	GCCGGATAACGGTGTACGCTGTATGTAATACAACCAGGAGGAAATCGATGCTGTGTAGGCTGGAGCTGCTTCG	
k2-EspO-del-Rv	GCAGACATCGGCACAATCCCTGTGGCGGTATCTGCCCGAAAAATCGTCAGGACATATGAATATCCTCCTTAGTTCC	
Ext-EspO-check-Fw	TGTATTGCATTAACAGGATCAC	
Ext-EspO-check-Rv	AGCTTTATGTATGTGTGCCTG	
EspA-CR-NcoI-Fw	CATGCCATGGATACATCAACTATGACATCAGTTGC	
EspA-CR-EcoRI-Rv	CGGAATTCGGTTTGCCAATGGGTATTGCTGAAACAG	

C. rodentium *espO* deletion mutant strain ICC1333 was constructed using the PCR one-step λ Red recombinase method (37). Briefly, the mutation was obtained using a PCR product containing the kanamycin resistance gene flanked by the 50 bases from the 5′ and 3′ ends of the targeted *espO* gene. Plasmid pKD4 was used as the PCR template with primers k1-EspO-del-Fw and k2-EspO-del-Rv (Table 1). The PCR product was electroporated into the recipient strain (wild-type C. rodentium [ICC169]) (Table 1) carrying the Red system expression plasmid pKD46, and mutants were selected on LB plates with kanamycin. Recombinant clones were cured of pKD46 plasmid by growth at the nonpermissive temperature (42°C) and mutation confirmed by PCR and DNA sequencing.

### Animals.

All animal experiments were performed in accordance with the Animals Scientific Procedures Act of 1986 and were approved by the local Ethical Review Committee. Pathogen-free female 18- to 20-g C57BL/6 mice were housed in HEPA-filtered cages with sterile bedding, food, and water.

Mice inoculated with the wild-type strain and/or with phosphate-buffered saline (PBS) (mock infection) were included in parallel with the mutant strain. A minimum of 5 and a maximum of 8 mice per group was used for each experiment. Each experiment was repeated a minimum of two times.

### Oral gavage of mice.

Mice were inoculated by oral gavage with 200 μl of overnight LB-grown C. rodentium suspension in PBS (∼5 × 10^9^ CFU) or mock infected with PBS. The number of viable bacteria used as the inoculum was determined by retrospective plating onto LB agar containing antibiotics. Stool samples were recovered aseptically at regular intervals after inoculation, and the number of viable bacteria per gram of stool was determined by plating onto LB agar. At appropriate time points, mice were culled and the colonic tissues collected for flow cytometry analysis. Cardiac punctures were performed for serum analysis.

For studies where mice were treated with antibiotics, at day 6 postinfection (p.i.) mice were gavaged without anesthesia and with 200 μl ciprofloxacin (100 mg/kg of body weight/day), chloramphenicol 100 mg/kg/day), or sterile distilled water (vehicle control) daily for 7 days.

### Sample collection for flow cytometry.

A 4-cm segment of the terminal colon was cut, opened longitudinally, and rinsed in sterile PBS. The tissue samples were placed in 4 ml of RPMI 1640 supplemented with 10% fetal bovine serum (FBS), penicillin-streptomycin (P/S), GlutaMAX, DNase (10104159001; Roche), and liberase (540112001; Roche) in a C-Mac tube (Miltenyi Biotec), followed by tissue dissociation using a gentleMACS dissociator (Miltenyi Biotec). First, the tissue was homogenized using the intestine setting followed by incubation at 37°C and 5% CO_2_ for 30 min in a shaking incubator, and then a final dissociation step was performed using the lung 2 setting on the gentleMACS dissociator. The digested preparation was disrupted to a single-cell suspension by passage through a 70-μm sieve (no. 352350; BD Labware/Falcon, USA) and resuspended in RPMI 1640 supplemented with 10% FBS and P/S at 0.5 × 10^6^ to 1 × 10^6^ cells/ml.

### Extracellular antigen analysis.

Cells were stained for surface markers (such as CD4^+^ CD8^−^ T cells, B220^+^ CD3^−^ B cells, and CD11b^+^ Ly6G^+^ neutrophils) in PBS containing 1% bovine serum albumin with 0.5% sodium azide (PBA) for 30 min at 4°C and fixed with IC fixation buffer (eBioscience) as described previously (31). Nonviable cells were excluded using a fixable near-infrared dead cell staining kit with excitation at 633 or 635 nm (L10119; Invitrogen). Antibodies were purchased from BD Pharmingen or eBioscience.

Data acquired on a BD Fortessa III with 20,000 live lymphocytes or myeloid events were analyzed with the FlowJo (TreeStar) analysis program. Data are shown as a percentage of live myeloid or lymphocyte gates. Myeloid and lymphocyte gates are determined by their position on the live/dead versus forward scatter plots generated by the cytometer. A fluorescence-minus-one (FMO) control was included for each fluorescent marker, and the expression of a particular marker was calculated by subtracting FMO fluorescence values from fluorescent antibody levels.

### Purification of recombinant C. rodentium EspA.

A pET28a-CR-EspA construct (pICC929) (Table 1), expressing C. rodentium EspA with a carboxyl-terminal 6-His affinity tag, was generated by PCR amplification from C. rodentium genomic DNA using primers EspA-CR-NcoI-Fw and EspA-CR-EcoRI-Rv (Table 1) and ligation of the NcoI- and EcoRI-digested amplicon into pET28a digested with the same enzymes. The resulting pICC929 plasmid was transformed into E. coli K-12 strain BL21(DE3) Star cells, which lack RNase E, to stabilize mRNA (Novagen), and the protein was expressed under isopropyl-β-d-thiogalactopyranoside (IPTG) induction and affinity purified with nickel resin using standard protocols. The yield, size, stability, and purity of the expressed protein was assessed by SDS-PAGE. Protein concentration was determined by the bicinchoninic acid method (Pierce Biotechnology, Inc., Rockford, IL, USA) (38).

### Detection of C. rodentium anti-EspA IgG in serum.

An enzyme-linked immunosorbent assay (ELISA) was used to detect serum IgG and antibodies to C. rodentium EspA. Sample dilutions were added to the antigen-coated wells (0.5 to 1 mg of C. rodentium EspA per well) of a microtiter 96-well plate. After incubation for 1 h at room temperature and subsequent washing, wells were incubated with horseradish peroxidase-conjugated donkey anti-mouse IgG. After further washing, tetramethylbenzidine substrate (TMB; Sigma) was added. Color formation was stopped after 10 min with 2.5 N sulfuric acid. The optical density was measured at 450 nm (OD_450_) with background correction at 620 nm. Fold change was calculated as the ratio to EspA IgG level detected in PBS mock-infected mice.

### *In vivo* optical imaging of C. rodentium-infected mice.

Whole-animal bioluminescence imaging (BLI) was performed daily following infection using an IVIS SpectrumCT (PerkinElmer) (39). In some studies, representative mice from each group were imaged using diffuse light imaging tomography with integrated μCT imaging (DLIT-μCT) as previously described (6, 39). At necropsy on either day 8 or 14 p.i., whole organs or washed excised gastrointestinal tissues with the mucosa exteriorized were imaged. Regions of interest were identified, and the total photon flux was quantified (photons/s) using Living Image software 4.3.1, service package 1 (PerkinElmer).

### Parameter inference.

For estimation of the parameters in our model (the bacterial growth rate, γ, neutrophil and B cell elimination rates, ν and β, and the factor for initial bacterial load, *f*), we implemented a probabilistic method called the Metropolis-Hastings algorithm, a Bayesian inference scheme. This is a Markov chain Monte Carlo method that enables sampling from the joint posterior distribution (21, 22); we used a Gaussian transition kernel to generate parameter proposals, normal priors for the growth rate and immune cell parameters, and a lognormal prior for the initial value.

We assumed additive Gaussian noise and used the measured variance in bacterial levels in the stool at each time point when calculating the likelihood. We linearly interpolated the immune cell populations at time points between measurements. We initialized the growth and immune cell parameters (γ, ν, and β) at a value of 1 with prior bounds (1e−3 and 1e3), and the initial value (*f*) at 1e−4 with prior bounds (1e−9 and 1). We performed 10 million iterations, removed a burn-in of 250,000 iterations, and thinned to every 5,000th iteration to give 1,950 sampled points from the posterior distribution. The posterior parameter distributions, means, and confidence intervals were calculated from the accepted parameter population.

### GP regression.

We use Gaussian process (GP) regression (23, 40), a nonparametric Bayesian method for nonlinear regression, to model the level of bacteria as a function of time using the experimental data. We used Matlab functions from the Gaussian Process Regression and Classification Toolbox, version 3.6 (23, 40), to infer values for the GP hyperparameters and fit the GP regression models; we assumed a zero-mean function and a squared covariance function and assumed the data are subject to normally distributed noise with constant variance.

### Statistics.

GraphPad Prism software was used for all statistical calculations. The Mann-Whitney test was used with PBS controls (or as indicated in the figure). *P* values of <0.05 were considered significant (*, *P* < 0.05; **, *P* < 0.01; ***, *P* < 0.001; ****, *P* < 0.0001).

## References

[1] Chen HD, Frankel G 2005 Enteropathogenic Escherichia coli: unravelling pathogenesis. FEMS Microbiol Rev 29:83–98. doi:10.1016/j.femsre.2004.07.002. 15652977

[2] Wong AR, Pearson JS, Bright MD, Munera D, Robinson KS, Lee SF, Frankel G, Hartland EL 2011 Enteropathogenic and enterohaemorrhagic Escherichia coli: even more subversive elements. Mol Microbiol 80:1420–1438. doi:10.1111/j.1365-2958.2011.07661.x. 21488979

[3] Hemrajani C, Berger CN, Robinson KS, Marchès O, Mousnier A, Frankel G 2010 NleH effectors interact with Bax inhibitor-1 to block apoptosis during enteropathogenic Escherichia coli infection. Proc Natl Acad Sci U S A 107:3129–3134. doi:10.1073/pnas.0911609106. 20133763 PMC2840288

[4] Pearson JS, Giogha C, Ong SY, Kennedy CL, Kelly M, Robinson KS, Lung TW, Mansell A, Riedmaier P, Oates CV, Zaid A, Mühlen S, Crepin VF, Marches O, Ang CS, Williamson NA, O'Reilly LA, Bankovacki A, Nachbur U, Infusini G, Webb AI, Silke J, Strasser A, Frankel G, Hartland EL 2013 A type III effector antagonizes death receptor signalling during bacterial gut infection. Nature 501:247–251. doi:10.1038/nature12524. 24025841 PMC3836246

[5] Li S, Zhang L, Yao Q, Li L, Dong N, Rong J, Gao W, Ding X, Sun L, Chen X, Chen S, Shao F 2013 Pathogen blocks host death receptor signalling by arginine GlcNAcylation of death domains. Nature 501:242–246. doi:10.1038/nature12436. 23955153

[6] Collins JW, Keeney KM, Crepin VF, Rathinam VAK, Fitzgerald KA, Finlay BB, Frankel G 2014 Citrobacter rodentium: infection, inflammation and the microbiota. Nat Rev Microbiol 12:612–623. doi:10.1038/nrmicro3315. 25088150

[7] Basu R, O'Quinn DB, Silberger DJ, Schoeb TR, Fouser L, Ouyang W, Hatton RD, Weaver CT 2012 Th22 cells are an important source of IL-22 for host protection against enteropathogenic bacteria. Immunity 37:1061–1075. doi:10.1016/j.immuni.2012.08.024. 23200827 PMC3678257

[8] Zheng Y, Valdez PA, Danilenko DM, Hu Y, Sa SM, Gong Q, Abbas AR, Modrusan Z, Ghilardi N, de Sauvage FJ, Ouyang W 2008 Interleukin-22 mediates early host defense against attaching and effacing bacterial pathogens. Nat Med 14:282–289. doi:10.1038/nm1720. 18264109

[9] Hoffmann C, Hill DA, Minkah N, Kirn T, Troy A, Artis D, Bushman F 2009 Community-wide response of the gut microbiota to enteropathogenic Citrobacter rodentium infection revealed by deep sequencing. Infect Immun 77:4668–4678. doi:10.1128/IAI.00493-09. 19635824 PMC2747949

[10] Willing BP, Vacharaksa A, Croxen M, Thanachayanont T, Finlay BB 2011 Altering host resistance to infections through microbial transplantation. PLoS One 6:e26988. doi:10.1371/journal.pone.0026988. 22046427 PMC3203939

[11] Ivanov II, Atarashi K, Manel N, Brodie EL, Shima T, Karaoz U, Wei D, Goldfarb KC, Santee CA, Lynch SV, Tanoue T, Imaoka A, Itoh K, Takeda K, Umesaki Y, Honda K, Littman DR 2009 Induction of intestinal TH17 cells by segmented filamentous bacteria. Cell 139:485–498. doi:10.1016/j.cell.2009.09.033. 19836068 PMC2796826

[12] Kamada N, Sakamoto K, Seo SU, Zeng MY, Kim YG, Cascalho M, Vallance BA, Puente JL, Núñez G 2015 Humoral immunity in the gut selectively targets phenotypically virulent attaching-and-effacing bacteria for intraluminal elimination. Cell Host Microbe 17:617–627. doi:10.1016/j.chom.2015.04.001. 25936799 PMC4433422

[13] Wiles S, Robertson BD, Frankel G, Kerton A 2009 Bioluminescent monitoring of in vivo colonization and clearance dynamics by light-emitting bacteria. Methods Mol Biol 574:137–153. doi:10.1007/978-1-60327-321-3_12. 19685306

[14] Wiles S, Pickard KM, Peng K, MacDonald TT, Frankel G 2006 In vivo bioluminescence imaging of the murine pathogen citrobacter rodentium. Infect Immun 74:5391–5396. doi:10.1128/IAI.00848-06. 16926434 PMC1594854

[15] Hall LJ, Murphy CT, Hurley G, Quinlan A, Shanahan F, Nally K, Melgar S 2013 Natural killer cells protect against mucosal and systemic infection with the enteric pathogen citrobacter rodentium. Infect Immun 81:460–469. doi:10.1128/IAI.00953-12. 23208605 PMC3553819

[16] Kherlopian AR, Song T, Duan Q, Neimark MA, Po MJ, Gohagan JK, Laine AF 2008 A review of imaging techniques for systems biology. BMC Syst Biol 2:74. doi:10.1186/1752-0509-2-74. 18700030 PMC2533300

[17] Ntziachristos V 2010 Going deeper than microscopy: the optical imaging frontier in biology. Nat Methods 7:603–614. doi:10.1038/nmeth.1483. 20676081

[18] Mei Y, Abedi V, Carbo A, Zhang X, Lu P, Philipson C, Hontecillas R, Hoops S, Liles N, Bassaganya-Riera J 2015 Multiscale modeling of mucosal immune responses. BMC Bioinformatics 16(Suppl 12):S2. doi:10.1186/1471-2105-16-S14-S2. PMC4705510 26329787

[19] Wendelsdorf KV, Alam M, Bassaganya-Riera J, Bisset K, Eubank S, Hontecillas R, Hoops S, Marathe M 2012 ENteric Immunity SImulator: a tool for in silico study of gastroenteric infections. IEEE Trans Nanobioscience 11:273–288. doi:10.1109/TNB.2012.2211891. 22987134 PMC3715318

[20] Kirk PD, Babtie AC, Stumpf MP 2015 Systems biology (un)certainties. Science 350:386–388. doi:10.1126/science.aac9505. 26494748

[21] Hastings WK 1970 Monte Carlo sampling methods using Markov chains and their applications. Biometrika 57:97–109. doi:10.1093/biomet/57.1.97.

[22] Wilkinson DJ 2012 Stochastic modelling for systems biology. CRC Press, Boca Raton, FL.

[23] Rasmussen CE, Williams CKI 2006 Gaussian processes for machine learning. MIT Press, Cambridge, MA.

[24] Kirk PD, Stumpf MP 2009 Gaussian process regression bootstrapping: exploring the effects of uncertainty in time course data. Bioinformatics 25:1300–1306. doi:10.1093/bioinformatics/btp139. 19289448 PMC2677737

[25] Liepe J, Filippi S, Komorowski M, Stumpf MP 2013 Maximizing the information content of experiments in systems biology. PLoS Comput Biol 9:e1002888. doi:10.1371/journal.pcbi.1002888. 23382663 PMC3561087

[26] Silk D, Kirk PD, Barnes CP, Toni T, Stumpf MP 2014 Model selection in systems biology depends on experimental design. PLoS Comput Biol 10:e1003650. doi:10.1371/journal.pcbi.1003650. 24922483 PMC4055659

[27] Lebeis SL, Bommarius B, Parkos CA, Sherman MA, Kalman D 2007 TLR signaling mediated by MyD88 is required for a protective innate immune response by neutrophils to citrobacter rodentium. J Immunol 179:566–577. doi:10.4049/jimmunol.179.1.566. 17579078

[28] Simmons CP, Clare S, Ghaem-Maghami M, Uren TK, Rankin J, Huett A, Goldin R, Lewis DJ, MacDonald TT, Strugnell RA, Frankel G, Dougan G 2003 Central role for B lymphocytes and CD4^+^ T cells in immunity to infection by the attaching and effacing pathogen citrobacter rodentium. Infect Immun 71:5077–5086. doi:10.1128/IAI.71.9.5077-5086.2003. 12933850 PMC187366

[29] Ogawa M, Fujita Y, Yoshikawa Y, Nagai T, Koyama T, Nagai S, Lange A, Fässler R, Sasakawa C 2009 Bacteria hijack integrin-linked kinase to stabilize focal adhesions and block cell detachment. Nature 459:578–582. doi:10.1038/nature07952. 19489119

[30] Morita-Ishihara T, Miura M, Iyoda S, Izumiya H, Watanabe H, Ohnishi M, Terajima J 2013 EspO1-2 regulates EspM2-mediated RhoA activity to stabilize formation of focal adhesions in enterohemorrhagic Escherichia coli-infected host cells. PLoS One 8:e55960. doi:10.1371/journal.pone.0055960. 23409096 PMC3568036

[31] Crepin VF, Habibzay M, Glegola-Madejska I, Guenot M, Collins JW, Frankel G 2015 Tir triggers expression of CXCL1 in enterocytes and neutrophil recruitment during Citrobacter rodentium infection. Infect Immun 83:3342–3354. doi:10.1128/IAI.00291-15. 26077760 PMC4534649

[32] Collins JW, Akin AR, Kosta A, Zhang N, Tangney M, Francis KP, Frankel G 2012 Pre-treatment with Bifidobacterium breve UCC2003 modulates Citrobacter rodentium-induced colonic inflammation and organ specificity. Microbiology 158:2826–2834. doi:10.1099/mic.0.060830-0. 22902730 PMC3541765

[33] Ale A, Ermolayev V, Herzog E, Cohrs C, Hrabe de Angelis M, Ntziachristos V 2012 FMT-XCT: *in vivo* animal studies with hybrid fluorescence molecular tomography-X-ray computed tomography. Nat Methods 9:615–620. doi:10.1038/nmeth.2014. 22561987

[34] Cronin M, Akin AR, Collins SA, Meganck J, Kim JB, Baban CK, Joyce SA, van Dam GM, Zhang N, van Sinderen D, O'Sullivan GC, Kasahara N, Gahan CG, Francis KP, Tangney M 2012 High resolution in vivo bioluminescent imaging for the study of bacterial tumour targeting, PLoS One 7:e30940. doi:10.1371/journal.pone.0030940. 22295120 PMC3266281

[35] Fallahi-Sichani M, El-Kebir M, Marino S, Kirschner DE, Linderman JJ 2011 Multiscale computational modeling reveals a critical role for TNF-α receptor 1 dynamics in tuberculosis granuloma formation. J Immunol 186:3472–3483. doi:10.4049/jimmunol.1003299. 21321109 PMC3127549

[36] Wiles S, Clare S, Harker J, Huett A, Young D, Dougan G, Frankel G 2004 Organ specificity, colonization and clearance dynamics in vivo following oral challenges with the murine pathogen citrobacter rodentium. Cell Microbiol 6:963–972. doi:10.1111/j.1462-5822.2004.00414.x. 15339271

[37] Datsenko KA, Wanner BL 2000 One-step inactivation of chromosomal genes in Escherichia coli K-12 using PCR products. Proc Natl Acad Sci U S A 97:6640–6645. doi:10.1073/pnas.120163297. 10829079 PMC18686

[38] Dziva F, Vlisidou I, Crepin VF, Wallis TS, Frankel G, Stevens MP 2007 Vaccination of calves with EspA, a key colonisation factor of Escherichia coli O157:H7, induces antigen-specific humoral responses but does not confer protection against intestinal colonisation. Vet Microbiol 123:254–261. doi:10.1016/j.vetmic.2007.02.016. 17374460

[39] Collins JW, Meganck JA, Kuo C, Francis KP, Frankel G 2013 4D multimodality imaging of Citrobacter rodentium infections in mice. J Vis Exp 78:50450. doi:10.3791/50450. PMC3855914 23979310

[40] Rasmussen CE, Nickisch H 2010 Gaussian processes for machine learning (GPML) toolbox. J Mach Learn Res 11:3011–3015.

